# Physical and Physiological Mechanisms of Emergent Hydrodynamic Pressure in High-Flow Nasal Cannula Therapy

**DOI:** 10.3390/arm94010001

**Published:** 2025-12-26

**Authors:** Jose Luis Estela-Zape

**Affiliations:** Faculty of Health, Universidad Santiago de Cali, Cali 760035, Colombia; jose.estela00@usc.edu.co or jose.estela55@gmail.com

**Keywords:** high-flow nasal cannula, positive-pressure respiration, respiratory mechanics, hydrodynamics, noninvasive ventilation, dead space

## Abstract

**Highlights:**

**What are the main findings?**

- HFNC generates transient, flow-dependent pressures, not sustained positive pressure, due to its open-system design and hydrodynamic principles.
- The primary therapeutic benefit of HFNC arises from dead-space washout and flow matching, not from pressure generation.

**What are the implications of the main findings?**

- Accurate terminology distinguishes HFNC as a flow-based system with secondary pressure effects, not a pressure modality; terms such as “emergent hydrodynamic pressure,” “flow-dependent transient pressure,” or “dynamic airway pressure” clarify this distinction.
- Individualized HFNC optimization should prioritize flow and patient anatomy over assumptions of pressure-driven effects.

**Abstract:**

High-flow nasal cannula (HFNC) therapy is frequently described as a positive pressure modality, yet this classification lacks mechanistic support. This critical narrative review integrates experimental, computational, and clinical evidence to examine the established physiological mechanisms underlying HFNC, with emphasis on precise terminology. The study clarifies that labeling HFNC as “positive pressure” is conceptually inaccurate, as the system delivers transient, flow-dependent pressures characteristic of open-circuit administration. Evidence is synthesized to quantify the relative contributions of nasopharyngeal dead-space clearance versus emergent pressure generation. Unlike CPAP, HFNC produces pressures ranging from 0.2 to 13.5 cmH_2_O, determined by airway geometry, leak magnitude, and mouth position. Fluid dynamic modeling using Bernoulli and Darcy–Weisbach equations demonstrates oscillatory rather than sustained pressures, with magnitudes linked to nasopharyngeal Reynolds numbers (2400–6000) and turbulent energy dissipation (30–60%). Clinical efficacy persists despite variable pressures, reflecting synergistic mechanisms: inspiratory flow matching (40–50% reduction in work of breathing), dead-space clearance (CO_2_ reduction, r = −0.77, *p* < 0.05), emergent pressure effects (10–20%), and thermal humidification (10–20%). Electrical impedance tomography reveals heterogeneous alveolar recruitment, with high-potential (54%) and low-potential (46%) phenotypes. Based on these mechanistic insights, this review proposes the term “emergent hydrodynamic pressure” to accurately describe HFNC’s transient, flow-dependent pressures. This terminology differentiates HFNC from conventional positive pressure systems and aligns language with the principles of fluid dynamics and respiratory physiology.

## 1. Introduction

High-flow nasal cannula (HFNC) therapy has been established as an effective form of noninvasive respiratory support in patients with acute hypoxemic respiratory failure. Its clinical application is associated with improved oxygenation, reduced breathing work, and better treatment tolerance compared with conventional oxygen delivery interfaces [1,2,3,4].

Despite extensive research into the physiology of high-flow nasal cannula (HFNC) therapy, terminology in clinical and research contexts remains inconsistent, as the terms “positive pressure” or “CPAP-like” combine mechanistically distinct phenomena. Although the fundamental mechanisms, including dead-space clearance, flow adaptation, and transient pressure generation, are identified, their relative contributions and conceptual framework remain poorly defined. This lack of standardization generates ambiguity between flow, pressure, and resistance, hindering accurate interpretation of HFNC’s physiological effects [5].

Unlike continuous positive airway pressure (CPAP), HFNC operates as an open system in which the generation of positive airway pressure is neither sustained nor represents its primary mechanism of action [4]. Experimental studies have shown that HFNC can generate transient end-expiratory airway pressures ranging from 2 to 10 cmH_2_O, modulated by flow rate, cannula diameter, oronasal leakage, and upper airway impedance. When the mouth is open, pressure may decrease by up to 75%, indicating that HFNC does not create a static positive pressure, but a dynamic phenomenon determined by flow conditions and airway anatomy. Therefore, equating HFNC with CPAP is conceptually inaccurate: CPAP provides continuous and regulated positive airway pressure within a closed circuit, whereas HFNC generates variable, flow-dependent pressures influenced by leaks and airway resistance [6,7,8].

The predominant physiological mechanism of HFNC is the washout of anatomical dead space, which facilitates the clearance of exhaled carbon dioxide (CO_2_) from the upper airways and reduces the volume of reinhaled gas. This flow- and time-dependent effect has been demonstrated in tracer gas studies showing a significant inverse correlation between delivered flow and reinhaled tracheal CO_2_ concentration (r = −0.77; *p* < 0.05) [9]. This mechanism consistently explains the reduction in breathing work compared with pressure generation itself [10,11]. Additionally, HFNC can produce transient positive pressures due to expiratory resistance against the gas jet and to flow impingement within the pharynx, where part of the kinetic energy (½ρv^2^) is converted into local static pressure [12,13]. Mechanical distension of the upper airway further reduces inspiratory resistance according to the Hagen–Poiseuille relationship (R ∝ 1/r^4^), decreasing the inspiratory effort required to achieve ventilatory flow [14,15].

From a hydrodynamic perspective, HFNC functions as an open system characterized by incompressible flow, governed by the balance between delivered flow, leak volume, and upper airway impedance. The kinetic energy of the gas dissipates mainly through turbulence within the nasopharynx (Reynolds number 2400–6000) and viscous friction along airway walls [16,17]. This explains why HFNC produces dynamic, transient pressures rather than sustained positive airway pressure, with magnitude depending on flow pattern, airway resistance, and individual anatomical features.

Thermal conditioning (31–37 °C) and active humidification of the gas, with relative humidity approaching 100%, are essential for optimal HFNC performance [18]. These factors reduce inspiratory resistance, preserve mucociliary function, and prevent reflex vasoconstriction of the nasal mucosa. Together with flow-related washout and decreased inspiratory effort, they account for the clinically observed reduction in work of breathing and diaphragmatic unloading. The absence of a linear correlation between generated pressure and reduction in work of breathing supports the hypothesis that HFNC benefits primarily arise from flow dynamics and airway resistance rather than from positive airway pressure per se [19,20].

The objective of this narrative review is to synthesize mechanistic evidence regarding HFNC physiology, reassess the conceptual accuracy of existing terminology, and propose refined terminology aligned with the physical principles governing HFNC function. This analysis integrates experimental data on pressure dynamics, computational modeling of airflow behavior, clinical measurements of work-of-breathing modulation, and evidence regarding alveolar recruitment mechanisms.

## 2. Physical–Physiological and Hydrodynamic Fundamentals of HFNC

### 2.1. Open-System Design and Flow Dynamics

HFNC is an open, non-sealed respiratory support system that delivers heated and humidified gas flows exceeding the patient’s inspiratory demand [11]. Unlike CPAP, HFNC does not impose fixed external pressure but generates a transient and variable emergent pressure resulting from the interaction between inspiratory flow demand, expiratory resistance, and leak dynamics within an open airway system. This distinction is fundamental to understanding HFNC not as a low-level CPAP device but as a dynamic flow–energy transfer interface governed by the principles of fluid mechanics and respiratory physiology [21].

From a hydrodynamic standpoint, airflow behavior in HFNC can be described through the conservation of mass and energy. The continuity Equation [10].

$$A_{1}v_{1}=A_{2}v_{2}$$

indicates that gas velocity varies inversely with the cross-sectional area. Consequently, anatomical narrowing of the nasopharynx or partial occlusion by the cannula prongs increases local flow velocity. However, the ideal Bernoulli Equation [6].

$$P+\frac{1}{2}\rho v^{2}+\rho gh=constant$$

is insufficient to characterize real flow conditions, as it assumes an inviscid, non-dissipative fluid. In HFNC, viscous and turbulent dissipation mechanisms, including wall shear stress (10–20 Pa at jet impingement zones), flow separation, and recirculation, account for approximately 30–60% of kinetic energy loss depending on airway anatomy and flow regime [22]. These dissipative effects transform part of the kinetic energy into heat and acoustic energy, producing spatially heterogeneous and transient pressure distributions observed in clinical settings [23,24].

### 2.2. Reynolds Number and Flow Regime

The flow regime in the upper airway is transitional to turbulent, with Reynolds numbers ranging from 2400 to 6000 in the nasopharyngeal cavity and 400 to 900 in the trachea, where the flow becomes progressively laminar [9]. Turbulent energy dissipation amplifies local pressure oscillations and contributes to the variability observed in nasopharyngeal pressure measurements [8,25]. The emergent pressure is therefore oscillatory rather than static, modulated by instantaneous flow phase and airway geometry [15].

### 2.3. Darcy–Weisbach Equation and Nonlinear Pressure–Flow Relationships

While Poiseuille’s law [12,26] describes laminar flow resistance, it is not applicable to HFNC conditions, where the flow regime is transitional or turbulent.

$$Q=\frac{\pi r^{4}(P_{1}-P_{2})}{8\eta L}$$

Under such conditions, resistance is better described by the Darcy–Weisbach Equation [27], where head loss is proportional to the square of flow velocity [17]. Consequently, the pressure–flow relationship becomes nonlinear, expressed as

$$Q\propto \sqrt{\Delta P}$$

for fully turbulent flow (Re > 4000). In transitional regimes (Re = 2400–4000) [18], the relationship is intermediate and depends on friction factors derived from Moody diagrams or iterative solutions of the Colebrook equation [28]. This nonlinearity explains why identical flow settings may yield markedly different nasopharyngeal pressures among individuals with variable airway morphology and leak dynamics.

Integrating these relationships, the emergent pressure *P*_e_ can be conceptually defined as:

$$P_{e}=f(Q_{in},Q_{out},R_{va},\alpha_{leak},A_{anatomy},E_{airway},t)$$

where *Q*_in_ and *Q*_out_ represent inspiratory and expiratory flow rates, *R*_va_ denotes airway resistance, α_leak_ is the effective leak area, *A*_anatomy_ reflects nasopharyngeal geometry, *E*_airway_ corresponds to airway compliance, and t indicates time dependence [29,30,31]. This formulation recognizes that HFNC pressure arises from complex flow–structure interactions rather than from externally applied pressure. The prong-to-nares occlusion ratio is a major determinant of pressure magnitude; ratios above 0.5 generate measurable positive pressures, whereas mouth opening can reduce them by 50–75% [2,32,33].

Physiologically, the primary therapeutic mechanism of HFNC is the washout of nasopharyngeal dead space, which reduces CO_2_ rebreathing and enhances alveolar ventilation. Computational and experimental studies report nasal cavity clearance half-times below 0.5 s at 45 L/min, with effective washout extending beyond the soft palate [11,21,34]. This rapid clearance decreases anatomical dead space across multiple airway compartments. A secondary mechanism involves inspiratory flow matching: when the delivered flow meets or exceeds the patient’s peak inspiratory demand (typically 30–60 L/min in acute hypoxemic respiratory failure), inspiratory effort and diaphragmatic workload decrease by up to 50%, as evidenced by reductions in transdiaphragmatic pressure and diaphragm electrical activity (EAdi) [35,36].

The emergent positive pressure produced by HFNC contributes to modest increases in end-expiratory lung volume (EELV), typically 2–5% in healthy subjects and up to 10–20% in patients with acute hypoxemia [5,37]. The extent to which this change represents alveolar recruitment or overdistension depends on baseline lung compliance and ventilatory pattern. Improvements in the ventilation–perfusion (V/Q) ratio are mainly attributed to dead-space reduction rather than correction of true shunt physiology [38].

Energy dissipation mechanisms, including turbulent friction, wall shear stress, flow separation, and downstream mixing, limit excessive pressure buildup and maintain system stability within physiological ranges. Continuous incoming flow during exhalation increases expiratory resistance, requiring patients to exhale against this positive pressure gradient. In patients with obstructive airway disease, such as COPD or asthma, with intact airway tone, this expiratory resistance can increase expiratory workload [31].

Conversely, in conditions associated with dynamic airway collapse, including tracheomalacia or severe expiratory airway collapse in bronchiectasis, the expiratory back-pressure provided by HFNC flow can help splint airways and prevent dynamic closure during exhalation. Bilevel HFNC systems, which modulate or reduce flow during exhalation, have been proposed to mitigate increased expiratory workload in patients with airway obstruction while preserving inspiratory flow benefits.

The thermal and humidification characteristics of HFNC are critical to its function. Delivery of fully humidified gas at 31–37 °C preserves mucociliary function, prevents reflex vasoconstriction, and minimizes inspiratory resistance, thereby improving comfort, compliance, and gas exchange efficiency [39,40]. Collectively, HFNC functions as an open energy transfer system in which flow-driven dynamics generate a transient hydrodynamically emergent pressure determined by resistance, geometry, and leak patterns. Understanding these physical and physiological interdependencies is essential to correctly interpret its mechanisms of action and to clarify that the so-called “positive pressure” in HFNC is not a constant imposed variable but an emergent property of the flow–resistance interaction within the upper airway [2,41].

## 3. Physiological Integration and Mechanistic Interpretation

### 3.1. Dead-Space Clearance Mechanisms

The physiological efficacy of HFNC therapy arises from the integrated effects of flow-dependent gas dynamics, upper airway clearance, inspiratory flow matching, and lung volume modulation. These mechanisms act synergistically to reduce dead-space rebreathing, decrease the work of breathing, and EELV, without relying on the simplified concept of continuous positive pressure [21,42].

Dead-space clearance is primarily convective, driven by the momentum of the delivered gas jet rather than by a passive flushing effect. Clearance kinetics are flow-dependent, with the half-time expired gas removal from the nasal cavity decreasing as HFNC flow increases:

$$t_{1/2}=f(Q_{HFNC})$$

Empirical observations show the following: 15 L/min → *t*_1/2_ ≈ 2–3 s, 30 L/min → *t*_1/2_ ≈ 1.2–1.5 s, and 45 L/min → *t*_1/2_ < 1 s [21,42,43]. The clearance rate is approximately linear in the anterior nasal cavity:

$${\overset{˙}{V}}_{clearance}\;=k\cdot Q_{HFNC},\;\;\;k\approx 1.8\;\mathrm{mL}/s\;\mathrm{per}\;L/\min$$

However, this linearity decreases markedly at depths beyond 2–3 cm (oropharynx). CO_2_ washout correlates strongly with flow in the anterior nasal cavity (R^2^ = 0.81), but correlation declines in the oropharynx (R^2^ ≈ 0.40–0.60) and deeper regions (R^2^ < 0.50), with minimal clearance below the soft palate [21,43,44]. Tracheal sampling demonstrates that inspired CO_2_ decreases with increasing HFNC flow:

$$FiCO_{2}=f(Q_{HFNC},Q_{out},\alpha_{leak})$$

with a strong negative correlation (r = −0.77, *p* < 0.05) and an approximate relationship:

$$\Delta (FiCO_{2})\approx -0.67\times \Delta Q_{HFNC}$$

At 45 L/min, inspired tracheal CO_2_ decreases by 30.2 mmHg at 2 cm depth, 14.9 mmHg at 3 cm, and 8.2 mmHg at 4 cm. Clearance efficiency depends strongly on airway geometry and compliance. The delivered jet’s kinetic energy is:

$$E_{kinetic}=\frac{1}{2}\rho_{gas}V_{jet}^{2}A_{jet}$$

with ρ_gas_ 1.2 kg/m^3^, *V*_jet_ ≈ 30–80 m/s at 60 L/min, and *A*_jet_ ≈ 12–33 mm^2^. Momentum dissipation limits effective clearance in deeper regions. Mouth position modulates both pressure and clearance: a closed mouth maximizes transient pressure but confines clearance, whereas an open mouth reduces nasopharyngeal pressure and paradoxically increases anatomical dead space [2,5,45].

### 3.2. Inspiratory Flow Matching and Work Breathing Reduction

Reduction in inspiratory work of breathing occurs through multiple mechanisms. The primary mechanism is flow-demand matching: when delivered flow approximates or exceeds the patient’s peak inspiratory flow ( $\overset{˙}{V}$ _HFNC_ ≥ $\overset{˙}{V}$ _patient_), the negative pressure gradient required to draw additional air is substantially reduced. The patient’s diaphragm generates pressure according to:

$$P_{diaphragm}={\overset{˙}{V}}_{patient}(R_{airway}+R_{tissue})+E_{system}V_{displaced}$$

When HFNC flow meets or exceeds demand, the required inspiratory pressure is reduced:

$$P_{diaphragm,HFNC}=({\overset{˙}{V}}_{patient}-{\overset{˙}{V}}_{HFNC})R_{total}+E_{system}V_{displaced}$$

Work of breathing (WOB) decreases nonlinearly with flow:

$$\Delta WOB=W_{max}(1-e^{-\lambda Q_{HFNC}})$$

where W_max_ ≈ 50% and λ ≈ 0.05–0.08 L/min^−1^ are estimated parameters [46]. Additionally, improved oxygenation with HFNC reduces ventilatory drive through feedback inhibition of respiratory centers, contributing to decreased work of breathing. Clinical studies report reductions of 30–50% in work of breathing at flows of 30–60 L/min, with limited additional benefit beyond 50 L/min, consistent with flow-dependent saturation described by Mauri et al. [5] and Pérez-Terán et al. [47]. The relative contributions of flow matching versus oxygenation-mediated ventilatory drive suppression depend on baseline hypoxemia severity and the trajectory of gas exchange improvement.

### 3.3. End-Expiratory Lung Volume and Alveolar Recruitment

HFNC produces a flow-dependent increase in end-expiratory lung volume, approximated by:

$$\Delta EELV=A\cdot (Q_{HFNC}-Q_{threshold})+baseline\;EELV$$

with *A* ≈ 0.003–0.005 L per L/min and Q_threshold_ ≈ 15–20 L/min. Electrical impedance tomography demonstrates heterogeneous regional recruitment and inflation dynamics. In supine patients [44], end-expiratory lung volume increases more in non-dependent (ventral) regions (+35% from baseline) than in dependent (dorsal) regions (+18%), whereas prone positioning produces a more uniform distribution. Globally, tidal volume remains stable or slightly decreases and preserved or improved compliance generally supports alveolar recruitment without overt global overdistension.

Regional heterogeneity, however, creates risk for selective overdistension of lower compliance–lower resistance (fast) compartments, even when global tidal volume is stable [43,46,48]. This overdistension may promote absorption atelectasis through accelerated nitrogen washout from poorly ventilated regions and could be harmful in conditions requiring protective ventilation strategies [43,46,48]. In some post-extubation cohorts, up to 36% of patients develop measurable regional overdistension on electrical impedance tomography despite stable global tidal volume, indicating that EELV and tidal volume metrics alone are insufficient to assess recruitment safety. These findings support the use of advanced monitoring, such as electrical impedance tomography or esophageal manometry, in patients at risk of regional overdistension.

Transient end-expiratory pressures (EEP) can be modeled as:

$$EEP(t)=P_{max}(1-e^{-t/\tau_{1}})-P_{leak}e^{-t/\tau_{2}}$$

with time constants τ_1_ ≈ 0.2–0.5 s, reflecting the transient buildup and decay of pressure.

### 3.4. Breathing Pattern and Airway Resistance Modification

HFNC reduces inspiratory airway resistance by 15 to 25% through jet-induced dilation and preservation of mucosal compliance. These mechanical effects, together with reduced work of breathing, produce consistent changes in breathing patterns: respiratory rate decreases by 3 to 5 breaths per minute, tidal volume remains stable or slightly decreases, and inspiratory and expiratory times are modestly prolonged [49,50].

The resulting slower and deeper breathing yields a more constant inspiratory flow profile compared with the high peak flows characteristic of tachypneic breathing. This shift promotes more homogeneous inflation of lung regions with heterogeneous compliance, preventing preferential inflation of higher-compliance areas over lower-compliance compartments that occur with high-velocity, peak inspiratory flows. Therefore, alveolar recruitment observed during HFNC therapy reflects not only pressure-induced distension but also flow-pattern modification that favors uniform ventilation distribution across lung compartments with different mechanical time constants.

### 3.5. Integrated Mechanistic Contribution

Overall, HFNC efficacy results from the combined contribution of multiple mechanisms. Flow matching and work reduction are dominant, dead-space washout contributes moderately, emergent pressure provides smaller benefit, and thermal and humidification effects add comfort and minor mechanical advantages (Table 1). Mechanistic analyses suggest that approximately 40–50% of WOB reduction derives from flow matching, 20–30% from dead-space washout, 10–20% from emergent pressure, and 10–20% from thermal and humidification effects, highlighting interindividual variability and the need for patient-specific assessment [31,45,51].

## 4. Critical Appraisal of Terminology: “Positive Pressure” vs. “Emergent Hydrodynamic Pressure”

The term positive pressure has become established in the clinical and research literature on HFNC therapy, appearing in foundational studies, clinical guidelines, and contemporary systematic reviews. Expressions such as positive pressure effect, PEEP-like, or CPAP-like are routinely used to describe HFNC’s physiological effects [21,22]. However, this terminology represents a conceptual error that conflates mechanically distinct phenomena and obscures the true physical and physiological basis of HFNC function. Historically, the term originated from the observation that HFNC systems generate measurable airway pressure. Although this is correct, it led to the mistaken extrapolation that HFNC operates as a positive pressure modality. Repeated citation and acceptance of this assumption have perpetuated an epistemological error, a case of semantic drift within respiratory literature [31,37,40].

HFNC generates pressure through mechanisms fundamentally different from CPAP. Although CPAP maintains pressure via active regulation by balancing circuit flow, inspiratory demand, and exhalation resistance, HFNC produces transient, flow-dependent pressure within an open system without active pressure control. The pressure generated by HFNC is physiologically relevant, typically ranging from 2 to 13.5 cmH_2_O depending on flow rate, patient-specific factors, and mouth position, but it arises because of flow dynamics rather than external regulation [12,22,35]. HFNC remains effective despite variable leaks through the mouth or around the cannula prongs, as this inherent leak is integral to its function as an open system. When leak pathways increase, such as with mouth opening, pressure decreases proportionally. However, the primary mechanisms, including dead-space clearance and flow matching, remain operative. The distinction between CPAP and HFNC is mechanistic rather than structural: CPAP sustains constant pressure through active regulatory feedback, whereas HFNC generates transient pressure as a secondary effect of flow interaction with airway anatomy and resistance [52,53,54].

HFNC pressure is transient rather than continuous. Maximum pressure occurs at end-expiration and rapidly dissipates during inspiration, approaching zero when patient inspiratory flow exceeds HFNC flow [22,52]. This behavior can be modeled as:

$$EEP(t)=P_{max}(1-e^{-t/\tau_{1}})-P_{leak}e^{-t/\tau_{2}},\tau_{1},\tau_{2}\approx 0.2-0.5\;s$$

This reflects that HFNC does not produce continuous positive pressure but transient oscillations. The pressure–flow relationship is not linear across all flow ranges: it is approximately linear in neonates but reaches a plateau at higher flows in adults. Nasopharyngeal pressure increases about 1.0–1.2 cmH_2_O per 10 L/min of flow with a closed mouth, but exhibits high interindividual variability, ranging from 0.2 to 13.5 cmH_2_O. This variability far exceeds that observed with CPAP, which maintains 3.5–9.9 cmH_2_O within comparable ranges, confirming that HFNC is inherently unpredictable compared with a sealed and regulated system [19,46,55,56,57].

The clinical efficacy of HFNC does not depend primarily on the pressure it generates. Even when mouth opening reduces pressure by 50–75%, work of breathing (WOB) reduction remains between 30% and 40%, indicating that the main mechanisms are flow-dependent: inspiratory flow matching and dead-space washout. Pressure is a secondary effect. The consistency of physiological response despite wide variability in pressure (0.2–13.5 cmH_2_O) supports the conclusion that pressure is not the primary causal mechanism of HFNC’s therapeutic benefits [11,58].

Mechanical comparison between HFNC and CPAP highlights essential differences. CPAP is a sealed and regulated system that provides sustained pressure, independent of flow, with low variability, and its principal mechanism is alveolar distension through pressure. HFNC, by contrast, is an open and unregulated system with transient, flow-dependent pressure (approximately 1 cmH_2_O per 10 L/min in adults, range 0.2–13.5 cmH_2_O). Its main effects are inspiratory flow-demand matching and dead-space washout, while pressure remains secondary. Mouth position and leak areas significantly affect HFNC pressure, unlike in CPAP, where their influence is minimal [57,59].

The term CPAP-like originally arose by analogy because HFNC generates measurable pressure and may promote alveolar recruitment in certain contexts. However, this analogy became institutionalized without critical reevaluation, perpetuating the mistaken belief that HFNC functions as a positive pressure system. This epistemological error leads to overestimation of the pressure contribution, underestimation of variability, and misinterpretation of clinical benefits and risks, hindering understanding of HFNC’s true mechanisms [38,42].

To address this conceptual ambiguity, we propose the term emergent hydrodynamic pressure as the primary descriptor, emphasizing that pressure arises as a secondary consequence of flow dynamics within an open system rather than as a regulated, primary therapeutic variable. This term reflects that [1] pressure is transient and secondary to flow, [2] its magnitude depends on multiple determinants, including flow, airway anatomy, and leak, and [3] pressure is not the primary mechanism driving therapeutic benefit.

Recognizing that “emergent hydrodynamic pressure” may not be immediately intuitive in clinical practice, alternative terms with equivalent mechanistic meaning include secondary pressure effects, flow-dependent transient pressure, or dynamic airway pressure. These alternatives highlight the distinction from CPAP’s regulated, sustained pressure while remaining clinically accessible.

Consequently, instead of the imprecise statement “HFNC generates PEEP-like pressure,” clinical communication should specify: “HFNC generates transient, flow-dependent pressures ranging from 0.2 to 13.5 cmH_2_O, determined by delivered flow, airway anatomy, and mouth position, with this pressure contributing approximately 10–20% to overall therapeutic effect” [50,58,60].

The clinical implications are clear. Flow optimization should be prioritized over assumptions of pressure generation. Mouth position and leaks must be actively managed. Device selection (HFNC vs. CPAP) should be based on the intended physiological mechanism, and individual evaluation is essential due to pressure heterogeneity. For research purposes, terminology should be standardized, mechanistic studies should decouple pressure from flow, biomarkers of response should be developed, and HFNC should be clearly classified as a flow-based support modality with secondary pressure effects [61,62].

Taken together, this analysis clarifies that the so-called “positive pressure” observed during HFNC therapy is not a regulated or sustained phenomenon but an emergent consequence of high-velocity flow within an open respiratory circuit. Recognizing this distinction shifts the interpretive framework of HFNC from pressure-driven to flow-driven physiology, aligning terminology with physical principles and clinical reality [58,63,64].

## 5. Clinical Implications and Patient-Specific HFNC Optimization

The physiological efficacy of HFNC arises from multiple interdependent mechanisms whose relative contributions vary substantially among patients. Optimal application requires integrating individual characteristics such as peak inspiratory flow demand, baseline work of breathing, airway geometry, body position, lung compliance, and overall clinical status into a dynamic titration strategy grounded in physiological principles [49,50]. The relationship between delivered flow and work of breathing reduction underscores the need for patient-specific assessment. The nonlinear saturation of benefit with increasing flow is described by:

$$\Delta WOB=W_{max}(1-e^{-\lambda Q_{HFNC}})$$

where W_max_ ≈ 50% represents the maximum achievable WOB reduction, and λ ≈ 0.05–0.08 L/min^−1^. Clinical studies show significant WOB reduction (30–50%) at flows of 30–60 L/min, with diminishing returns beyond 50 L/min [22].

The primary determinant of optimal HFNC flow is the relationship between the patient’s peak inspiratory flow (PIF) and the delivered flow. In healthy individuals and patients with mild-to-moderate hypoxemic respiratory failure, PIF typically ranges from 30 to 60 L/min. In critically ill patients with acute respiratory distress or elevated respiratory drive, PIF often exceeds 100 L/min and can reach 200–300 L/min or higher [62,65,66,67]. When flow meets or exceeds PIF, the inspiratory pressure gradient is reduced, maximizing work-of-breathing reduction. If delivered flow falls substantially below PIF, the flow-matching benefit decreases.

Clinically, HFNC flow is initiated at moderate-to-high levels—typically 20 to 50 L/min in stable patients, with higher initial flows in critically ill patients with elevated PIF—while continuously monitoring respiratory pattern, comfort, oxygenation, and inspiratory effort. Flow is titrated in 5–10 L/min increments until a physiological plateau is reached, usually 30–60 L/min in most patients. Excessive flows above 60 L/min may increase end-expiratory lung volume and the risk of regional overdistension without additional benefit, particularly in patients with normal PIF; however, flows above 60 L/min may be indicated in critically ill patients to match elevated PIF demands [68,69].

EEP increases with HFNC flow according to the transient model:

$$EEP(t)=P_{max}(1-e^{-t/\tau_{1}})-P_{leak}e^{-t/\tau_{2}}$$

where τ_1_ ≈ 0.2–0.5 s.

In clinical practice, HFNC flow titration should be guided by physiological parameters and patient-specific responses rather than fixed or universal protocols. Flows are generally initiated at intermediate levels (approximately 20–50 L/min) and adjusted in 5–10 L/min increments while continuously monitoring respiratory rate, breathing pattern, comfort tolerance, oxygenation, and perceived inspiratory effort. The operational goal is to match or exceed the patient’s peak inspiratory flow to achieve measurable reductions in work of breathing without generating indicators of regional overdistension, such as unexplained increases in end-expiratory lung volume or adverse patterns on electrical impedance tomography. Very high flows (≥60 L/min) may produce disproportionate increases in EELV without corresponding improvements in work of breathing or oxygenation; therefore, their application should be individualized according to physiological response [58,69].

EEP generation is highly variable, ranging from 0.2 to 6 cmH_2_O with the mouth closed and 0.2 to 1.3 cmH_2_O with the mouth open at 60 L/min, depending on anatomical factors. Nasopharyngeal pressure increases approximately 1.0–1.2 cmH_2_O per 10 L/min of flow under closed-mouth conditions. Prong-to-nares occlusion ratios greater than 0.5 enhance pressure generation, whereas ratios below 0.5 minimize excessive pressure accumulation. Because of the high interindividual variability, pressure should not be inferred from flow settings alone but verified at the bedside. Inspiratory airway resistance decreases by 15–25% through jet-induced mechanical dilation and humidification preservation, with minimal further reduction at flows above 40 L/min [2,24,70,71].

Electrical impedance tomography (EIT) demonstrates position-dependent alveolar recruitment. Recent studies in post-extubation patients (*n* = 24) identified two response phenotypes: high-potential-recruitment (HPR, 54%, *n* = 13) and low-potential-recruitment (LPR, 46%, *n* = 11). HPR patients exhibited recruitment without overdistension across flows of 20–60 L/min, while LPR patients showed either no aeration change (7 patients, 29%) or regional overdistension (4 patients, 17%) despite stable global tidal volume. This heterogeneity highlights the limitations of global metrics in predicting individual HFNC responses and supports the use of advanced monitoring tools (EIT or esophageal manometry) for individualized optimization [72,73].

The ROX index [74], defined as:

$$ROX=\frac{SpO_{2}/FiO_{2}}{RR}$$

serves as a bedside predictor of HFNC success or failure. Thresholds vary according to clinical population and context. The original cutoff of ROX ≥ 4.88 at 12 h was validated in ICU patients with pneumonia; subsequent studies reported higher thresholds in other cohorts, including ROX ≥ 5.99 in COVID-19 pneumonia, ROX ≥ 5.35 in severe community-acquired pneumonia (CAP), and ROX ≥ 14.79 in non-ICU hospitalized patients. These thresholds should be interpreted within the context of the specific patient population rather than applied universally [3,75,76,77,78].

Early identification of HFNC failure is essential to prevent delayed endotracheal intubation. Among available predictors, the ROX index functions as a dynamic bedside metric with validated performance. The original cutoff (ROX ≥ 4.88 at 12 h) was established in ICU patients with pneumonia, whereas subsequent studies have reported higher thresholds in other clinical settings, including ROX ≥ 5.99 in COVID-19 pneumonia, ROX ≥ 5.35 in severe community-acquired pneumonia, and ROX ≥ 14.79 in non-ICU hospitalized patients. These thresholds should be interpreted within the context of the specific population and clinical condition, rather than applied as universal criteria [76,79].

Baseline lung compliance combined with dynamic oxygenation trends provides superior predictive accuracy for HFNC failure. Compliance < 30 mL/cmH_2_O with a PaO_2_/FiO_2_ change < 20% at 24 h yields an area under the curve (AUC) of 0.88, outperforming ROX alone. Compliance alone (AUC 0.82) and baseline compliance < 30 mL/cmH_2_O (OR 3.52, 95% CI 1.92–6.45), or ΔPaO_2_/FiO_2_ < 20% at 24 h (OR 2.84, 95% CI 1.48–5.43), are also strong independent predictors [79,80].

HFNC failure risk increases with age ≥ 70 years (HR 3.4), presence of stroke as a comorbidity (OR 2.48), chronic kidney disease, and low baseline SOFA score. Early warning signs (1–4 h) include SpO_2_/FiO_2_ < 150–200 and ROX < 2.85 at 1 h, both with > 80% specificity for intubation. Evolving indicators (6–12+ h) include a declining ROX index, decreasing compliance, and evidence of regional overdistension on EIT. Optimal HFNC use integrates patient physiology, mechanism-specific contributions, positional effects, and continuous physiological monitoring. A dynamic titration approach rather than fixed, protocol-driven flows accommodates interindividual variability and enables mechanism-targeted optimization, thereby maximizing therapeutic efficacy while minimizing overdistension and HFNC failure [63,72,75,79].

### Physiological Limitations and Appropriate Clinical Indications

The mechanistic characterization of HFNC as a primarily flow-based system with secondary pressure effects defines its clinical role and limitations. HFNC is limited in scenarios requiring sustained elevation of mean airway pressure or substantial inspiratory pressure support. In acute respiratory distress syndrome (ARDS) and conditions with severely reduced lung compliance, the transient pressures generated by HFNC (0.2–13.5 cmH_2_O) are insufficient to achieve the alveolar recruitment attainable with CPAP or bilevel noninvasive ventilation (8–15 cmH_2_O). Similarly, in COPD exacerbations requiring significant inspiratory pressure, HFNC cannot provide the active support delivered by pressure-assisted ventilation modes.

HFNC is most effective in contexts where high FiO_2_ delivery is essential and sustained airway pressure is either unnecessary or potentially harmful. These include hypoxemic respiratory failure in restrictive lung diseases, pulmonary hypertension with hypoxemia, hypoxemic respiratory failure in patients with marginal hemodynamics, and post-extubation support in patients with near-normal lung mechanics. In these cases, HFNC’s primary mechanisms (dead-space clearance, flow matching, and oxygenation delivery) provide the intended physiological effect, while secondary pressure contributions remain modest.

Clinical selection between HFNC and other noninvasive modalities should be guided by underlying physiology. When oxygenation improvement and reduction in work of breathing are the primary goals without the need for sustained airway pressure, HFNC is appropriate. If sustained mean airway pressure or active inspiratory support is required, CPAP or bilevel ventilation should be used. This approach targets HFNC use in situations where its mechanistic properties are physiologically relevant.

## 6. Methodological Approach and Literature Integration

This critical narrative review integrates experimental, computational, and clinical evidence to analyze the physical and physiological mechanisms underlying emergent hydrodynamic pressure in HFNC therapy, with emphasis on differentiating HFNC from conventional positive pressure systems.

Literature selection prioritized peer-reviewed original studies, systematic reviews, and clinical guidelines addressing HFNC mechanisms, including nasopharyngeal pressure behavior, dead-space clearance, work-of-breathing modulation, and predictors of clinical response or failure. Specific physical variables were analyzed in detail, including airway geometry, inspiratory flow rates, nasopharyngeal pressures, Reynolds numbers, and turbulent energy dissipation, providing a mechanistic interpretation grounded in fluid dynamics. Mechanistic findings were then integrated with physiological outcomes, linking oscillatory pressures, flow-dependent effects, and dead-space washout to reductions in work of breathing, alveolar recruitment patterns, and CO_2_ clearance.

The methodology allowed clear differentiation between HFNC and CPAP mechanisms and supported quantification of the relative contributions of flow, transient pressure, and dead-space clearance, forming the basis for the proposed terminology of emergent hydrodynamic pressure.

## 7. Conclusions

Characterizing HFNC as a positive pressure modality represents a persistent epistemological error. Physical evidence, including flow-dependent behavior, open-system geometry, transient pressure dynamics, and comparison with CPAP, combined with physiological findings demonstrating therapeutic effects despite variable pressures, confirms that conventional terminology lacks a mechanistic foundation. Adopting the concept of emergent hydrodynamic pressure provides a mechanistic framework that accurately describes HFNC function, differentiating it from pressure-regulated systems such as CPAP, and informs patient-specific clinical application. It emphasizes that HFNC efficacy arises primarily from flow-dependent mechanisms—dead-space clearance, flow matching, and oxygenation delivery—rather than sustained airway pressure.

This framework also guides future research, including the identification of biomarkers predicting individual response, assessment of flow-pattern effects independent of pressure, and optimization of flow and thermal humidification parameters. Establishing mechanistic clarity enhances both the precision of clinical application and the design of physiologically grounded studies on HFNC efficacy.

## Figures and Tables

**Table 1:** Pressure dynamics and mechanistic differences between HFNC and CPAP.

Characteristic	HFNC	CPAP	Mechanistic Implication
System design	Open, non-sealed	Closed, sealed	CPAP needs a seal; HFNC works despite leaks
Source of pressure	Flow–resistance (emergent)	Actively regulated	HFNC pressure is secondary; CPAP is controlled
Pressure profile	Transient, flow-dependent	Sustained, regulated	HFNC cannot maintain continuous pressure
Pressure stability	Variable	Minimal variability	CPAP predictable; HFNC varies with leaks
Primary mechanism	Dead-space washout + flow relief	Continuous distending pressure	HFNC is kinetic; CPAP is static pressure-driven
Secondary mechanism	Emergent hydrodynamic distending pressure	Pressure-mediated recruitment	Pressure minor in HFNC; central in CPAP
Effect of mouth opening	Reduces pressure 50–75%	Minimal impact	Oral leak affects HFNC; CPAP maintains effect
Inter-subject variability	High	Low	HFNC responses vary; CPAP is consistent
Reduction in work of breathing	30–50% (flow)	40–60% (pressure)	Similar effect via different mechanisms
Flow/pressure–effect consistency	Effect preserved	Correlates with pressure	HFNC depends on flow; CPAP on pressure
Flow–pressure relationship	Nonlinear, leak-dependent	N/A	HFNC follows fluid dynamics; CPAP is set pressure
Energy dissipation	Partial via turbulence	N/A	Limits peak pressures in HFNC
Operational target	Flow ≥ demand (≈30–60 L/min)	Fixed pressure (≈4–8 cmH_2_O)	HFNC titrated by flow; CPAP by pressure
Predictors of response	ROX, ventilatory pattern	Compliance, clinical status	HFNC phenotype-dependent; CPAP standardized
Risk of overdistension	Possible in low-reserve groups	Low	HFNC may need monitoring; CPAP safer

## Data Availability

The authors declare that all data supporting the report are available upon request from the corresponding author.

## Abbreviations

The following abbreviations are used in this manuscript:

HFNC	High-flow nasal cannula
CPAP	Continuous positive airway pressure
EELV	End-expiratory lung volume
WOB	Work of breathing
EAdi	Diaphragm electrical activity
PIF	Peak inspiratory flow
EEP	End-expiratory pressure
ROX	Ratio of oxygen saturation to respiratory rate
AUC	Area under the curve
OR	Odds ratio
HR	Hazard ratio
CI	Confidence interval
PaO_2_/FiO_2_	Ratio of arterial oxygen partial pressure to fraction of inspired oxygen
SpO_2_/FiO_2_	Ratio of peripheral capillary oxygen saturation to fraction of inspired oxygen
EIT	Electrical impedance tomography
HPR	High-potential-recruitment
LPR	Low-potential-recruitment
PEEP	Positive end-expiratory pressure
V/Q	Ventilation/perfusion
SOFA	Sequential Organ Failure Assessment
Pa	Pascal
R^2^	Coefficient of determination
r	Correlation coefficient
